# The predictive value of the lactate dehydrogenase-to-albumin ratio for mortality in patients with septic shock

**DOI:** 10.1097/MD.0000000000046981

**Published:** 2026-01-02

**Authors:** Yin-Yin Wu, Li Yao

**Affiliations:** a Intensive Care Unit, The Second People’s Hospital of Hefei, Hefei Hospital Affiliated to Anhui Medical University, Hefei City, Anhui Province, China.

**Keywords:** albumin, lactate dehydrogenase-to-albumin ratio, sepsis, septic shock

## Abstract

Septic shock is a life-threatening condition with high mortality despite advances in intensive care. Identifying reliable and easily obtainable biomarkers is essential for early risk stratification. Lactate dehydrogenase-to-albumin ratio (LAR), which combines 2 routine laboratory tests, has recently been suggested as a prognostic marker in sepsis. To investigate the predictive value of LAR for 28-day mortality in patients with septic shock. This retrospective study included 93 patients with septic shock admitted to the intensive care unit of the Second People’s Hospital of Hefei. Patients were categorized into survival (n = 41) and non-survival (n = 52) groups based on their 28-day outcomes. Univariate and multivariate analyses were performed to compare demographics, clinical indicators, and treatments. A restricted cubic spline (RCS) model was used to assess the relationship between LAR and mortality. Patients were stratified into high- and low-LAR groups using the optimal cutoff identified by the RCS model. Kaplan–Meier survival curves and the log-rank test were used to compare 28-day survival between groups. Univariate analysis revealed significant differences between the survival and non-survival groups in terms of age, acute physiology and chronic health evaluation II score, Sequential Organ Failure Assessment score, intensive care unit length of stay, LAR, blood urea nitrogen, lactic acid, white blood cell count, mechanical ventilation, and norepinephrine use. Multivariate analysis identified LAR and white blood cell count as independent risk factors for mortality. RCS analysis demonstrated a nonlinear relationship between LAR and all-cause mortality, with a hazard ratio of 1 at a LAR value of 10.1. Patients in the high-LAR group (≥10.1) had significantly lower 28-day cumulative survival compared to those in the low-LAR group (<10.1; *P* = .011). LAR is an independent predictor of 28-day mortality in patients with septic shock, with higher values associated with increased risk. Patients with elevated LAR (≥10.1) should be closely monitored. However, as this was a retrospective single-center study with a relatively small sample size, the findings should be interpreted cautiously, and larger prospective studies are warranted.

## 1. Introduction

Sepsis is a life threatening condition characterized by organ dysfunction resulting from a dysregulated host response to infection.^[1]^ When sepsis progresses to septic shock, defined by persistent hypotension requiring vasopressors and elevated lactate levels despite adequate fluid resuscitation, the risk of mortality increases substantially. Septic shock remains a major challenge in critical care medicine, with 28 day mortality rates ranging from 30% to 50% depending on disease severity, patient characteristics, and available healthcare resources.^[1–3]^ Early identification of patients at high risk is essential for timely intervention and improved clinical outcomes.

Lactate dehydrogenase (LDH) is an intracellular enzyme that is released into the circulation during tissue damage, hypoxia, and cellular stress – all common occurrences in sepsis and septic shock. Elevated LDH levels have been associated with the development of organ failure and poor prognosis in critically ill patients.^[2,4–6]^ Albumin (ALB), on the other hand, is a plasma protein synthesized in the liver and serves as a marker of both nutritional status and systemic inflammation. Low albumin levels are frequently observed in septic patients and have been linked to worse outcomes.^[6,7]^ The lactate dehydrogenase-to-albumin ratio (LAR) combines these 2 routinely measured biomarkers and has recently gained attention as a potential predictor of mortality in sepsis. Several studies using data from the Medical Information Mart for Intensive Care IV (MIMIC IV) database in the United States have suggested that LAR is an independent predictor of 28 day mortality in patients with septic shock.^[2–5]^

However, the applicability of these findings to non Western populations remains unclear. Differences in genetic background, environmental exposures, disease burden, and healthcare practices may influence sepsis outcomes across populations. Currently, limited data are available regarding the prognostic value of LAR in Chinese patients with septic shock. Therefore, this study aimed to evaluate the association between LAR and 28 day mortality in patients with septic shock admitted to the intensive care unit (ICU) of the Second People’s Hospital of Hefei. By exploring this readily available biomarker in a real world clinical setting, we hope to provide evidence to support its use in early risk stratification and individualized management. Despite extensive research, progress in septic shock management has been limited. The heterogeneity of the condition and challenges in clinical research highlight the need for biomarkers that are specific, prognostically meaningful, and easily available in routine practice. Identifying such markers can help clinicians intervene earlier and guide individualized treatment strategies.

## 2. Material and methods

### 2.1. Data source

**Figure 1.**
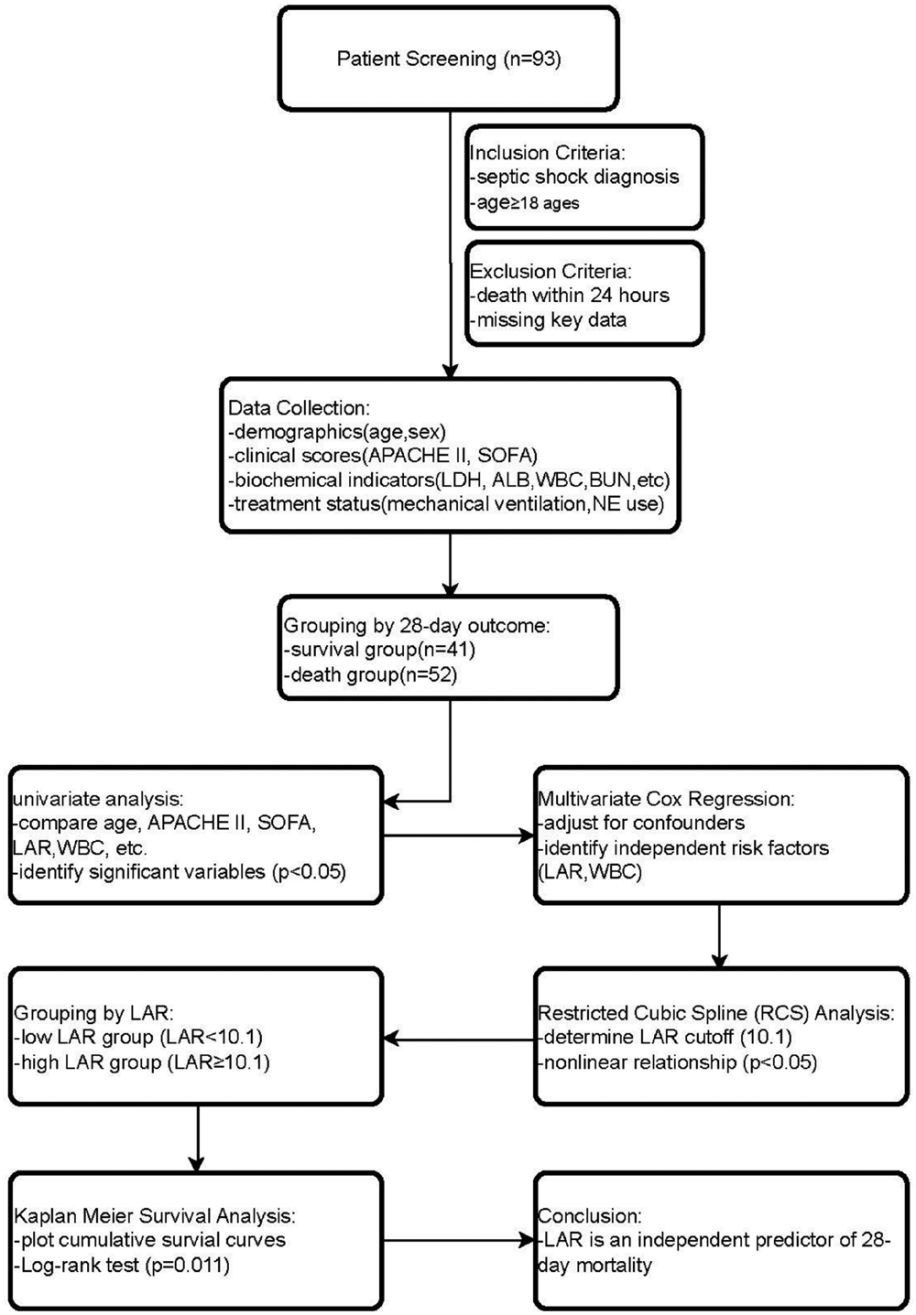
The flow chart of the study. ALB = albumin, APACHE II = Acute Physiology and Chronic Health Evaluation II, BUN = blood urea nitrogen, LAR = lactate dehydrogenase-to-albumin ratio, LDH = lactate dehydrogenase, RCS = restricted cubic spline, SOFA = Sequential Organ Failure Assessment, WBC = white blood cell.

In this study, a retrospective research method was used. This study was conducted according to the guidelines of the Declaration of Helsinki, and approved by the Institutional Review Board of the Second People’s Hospital of Hefei City. The included patients were patients with septic shock admitted to the ICU of the Second People’s Hospital of Hefei between January 2021 and January 2024. The inclusion criteria were as follows: septic shock was diagnosed according to The Third International Consensus Definitions for Sepsis and Septic Shock and patients were aged ≥ 18 years. The exclusion criteria were as follows: death within 24 hours of admission to the ICU or missing data on key missing data on key variables such as the LAR. The flow chart of the study is shown in Figure 1.

### 2.2. Methods

The following general data of the patients were collected at ICU admission (within the first 24 hours): age, sex, underlying diseases, Sequential Organ Failure Assessment (SOFA) score, Acute Physiology and Chronic Health Evaluation II (APACHE II) score; clinical indicators such as LDH, ALB, blood urea nitrogen (BUN), creatinine, lactic acid, alanine aminotransferase, γ-glutamyltransferase, total bilirubin, blood glucose, white blood cells (WBCs), hemoglobin, platelets, serum potassium, serum magnesium, serum phosphorus, total serum calcium, international normalized ratio, and fibrinogen; treatment status (mechanical ventilation, continuous renal replacement therapy, norepinephrine use, and plasma transfusion), and ICU length of stay. Norepinephrine was recorded only as a binary variable (yes/no), as dose and timing were not available in the medical records.

The patients were divided into a survival group and a mortality group according to the 28-day survival. The best cutoff value was obtained via restricted cubic spline (RCS) analysis, and the patients were divided into a low-LAR group (LAR < 10.1) and a high-LAR group (LAR ≥ 10.1). The primary objective of this study was to evaluate the association between LAR and 28-day mortality in patients with septic shock. Secondary objectives were to explore the impact of WBC count and other clinical covariates on mortality. The study outcome indicator was 28-day mortality.

### 2.3. Statistical methods

Stana 15.1 software was used for data analysis. Normally distributed continuous variables are expressed as the mean ± standard deviation, and a *T* test was performed; nonnormally distributed continuous variables are expressed as the median (interquartile range), and the rank test was performed. Categorical variables are expressed as percentages, and the *x*^2^ test was performed. The overall association between the LAR and risk of 28-day mortality in patients with septic shock was analyzed via RCS. The Kaplan–Meier survival curve and log-rank test were used to compare the difference in 28-day cumulative survival between the low-LAR and high-LAR groups. Multivariate Cox regression analysis was used to analyze the influencing factors. *P* < .05 indicated that the difference was statistically significant.

## 3. Results

### 3.1. General characteristics

A total of 93 patients with septic shock were enrolled, of whom 52 (55.9%) died within 28 days. Compared to the survival group (n = 41), the mortality group had significantly higher age (75.36 vs 68.74 years), APACHE II score (27 vs 22), SOFA score (11.69 vs 8.39), ICU length of stay (14 vs 6 days), LAR (10.13 vs 6.88), lactic acid (4.53 vs 2.4 mmol/L), BUN (17.9 vs 16.1 mmol/L), and WBC count (18.37 vs 9.57 × 10⁹/L; all *P* < .05; Table 1). The use of mechanical ventilation (94.2% vs 58.5%) and norepinephrine (90.4% vs 51.2%) was also significantly more frequent in the mortality group (*P* < .05). No significant differences were observed in LDH or ALB levels alone.

**Table 1:** Comparison of the general information, clinical indicators, and treatment of the patients in the 2 groups.

Item	Survival group (n = 41)	Mortality group (n = 52)	*T*/*x*^2^/*z* value	*P* value
Age (yr)	68.74 ± 10.99	75.36 ± 12.93	−2.27	<.05
Male (case [%])	21 (51.22)	27 (51.92)	0.259	.611
SOFA score (points)	8.39 ± 2.80	11.69 ± 4.33	−3.68	<.05
APACHE II score (points)	22 (18, 25)	27 (20, 30)	−2.893	<.05
ICU length of stay (d)	6 (2, 9)	14 (7, 21)	3.464	<.05
Biochemical indicators				
ALB (g/L)	29.95 ± 6.44	28.65 ± 5.97	0.872	.193
LDH (U/L)	263 (210, 333)	259 (185, 478)	−0.367	.714
LAR	6.88 (6.11, 9.53)	10.13 (7.91, 18.71)	−2.962	<.05
Lactic acid (mmol/L)	2.4 (1.53, 5.55)	4.53 (3.23, 7.4)	−3.151	<.05
BUN (mmol/L)	16.1 (5.7, 2.19)	17.9 (12.2, 39.4)	−2.128	<.05
Creatinine (mmol/L)	166.9 (67,299)	173.4 (82.8303.1)	−0.136	.892
ALT (U/L)	39 (17, 72)	35 (18, 63)	0.025	.980
GGT (U/L)	34 (20,111)	32 (18,76)	0.754	.451
Total bilirubin (mmol/L)	16.2 (10.9, 22.2)	15.2 (10.7, 26.8)	−0.124	.91
Blood glucose (mmol/L)	8.75 (5.48, 11.07)	9.86 (6.52, 11.92)	−1.035	.300
WBC (×10^9^/L)	9.57 (5.3, 15.5)	18.37 (10.49, 25.19)	2.796	<.05
Hemoglobin (g/L)	107.71 ± 27.64	96.76 ± 29.59	1.584	.059
Platelet (×10^9^/L)	189 (105, 279)	153 (59, 230)	1.419	.156
Serum potassium (mmol/L)	4.01 (3.65, 4.58)	4.46 (3.61, 5.03)	−1.395	.163
Serum magnesium (mmol/L)	0.87 ± 0.25	0.86 ± 0.27	0.138	.445
Serum phosphorus (mmol/L)	1.21 (1.06, 1.55)	1.49 (1.04, 2.36)	−1.102	.271
Total serum calcium (mmol/L)	2.02 (1.9, 2.14)	2.03 (1.88, 2.14)	−0.03	.976
Serum ionized calcium (mmol/L)	1.14 (1.07, 1.22)	1.12 (1.04, 1.23)	0.345	.73

ALB = albumin, ALT = alanine aminotransferase, APACHE II = Acute Physiology and Chronic Health Evaluation II, BUN = blood urea nitrogen, CRRT = continuous renal replacement therapy, GGT = γ-glutamyltransferase, ICU = intensive care unit, INR = international normalized ratio, LAR = lactate dehydrogenase-to-albumin ratio, LDH = lactate dehydrogenase, NE = norepinephrine, SOFA = Sequential Organ Failure Assessment, WBC = white blood cell.

### 3.2. Multivariate analysis

Multivariate Cox regression identified LAR (*P* = .043) and WBC count (*P* = .026) as independent predictors of 28-day mortality. Other variables, including age, APACHE II score, SOFA score, and norepinephrine use, showed marginal significance but did not reach statistical thresholds (Table 2).

**Table 2:** Multivariate analysis results.

	Chef	SE	Wald *Z*	*P*(> *Z* )
Age	0.063	0.050	1.260	.207
APACHE II score (points)	0.337	0.181	1.860	.062
SOFA score (points)	−0.494	0.286	−1.730	.084
LAR	0.478	0.236	2.030	.043
Lactic acid (mmol/L)	0.274	0.225	1.220	.223
Mechanical ventilation = yes	9.355	5.317	1.760	.079
NE use = yes	2.603	1.690	1.540	.123
ICU length of stay (d)	−0.186	0.099	−1.890	.059
WBC (×10^9^/L)	−0.237	0.107	−2.220	.026
BUN (mmol/L)	0.175	0.111	1.570	.116

APACHE II = Acute Physiology and Chronic Health Evaluation II, BUN = blood urea nitrogen, ICU = intensive care unit, LAR = lactate dehydrogenase-to-albumin ratio, NE = norepinephrine, SE = standard error, SOFA = Sequential Organ Failure Assessment, WBC = white blood cell.

### 3.3. Association between LAR and mortality

RCS analysis revealed a nonlinear association between LAR and 28-day all-cause mortality (*P* for overall = .002; *P* for nonlinearity = .910). A LAR value of 10.1 corresponded to a hazard ratio of 1, indicating this threshold as an optimal cutoff.

### 3.4. Survival analysis

**Figure 2.**
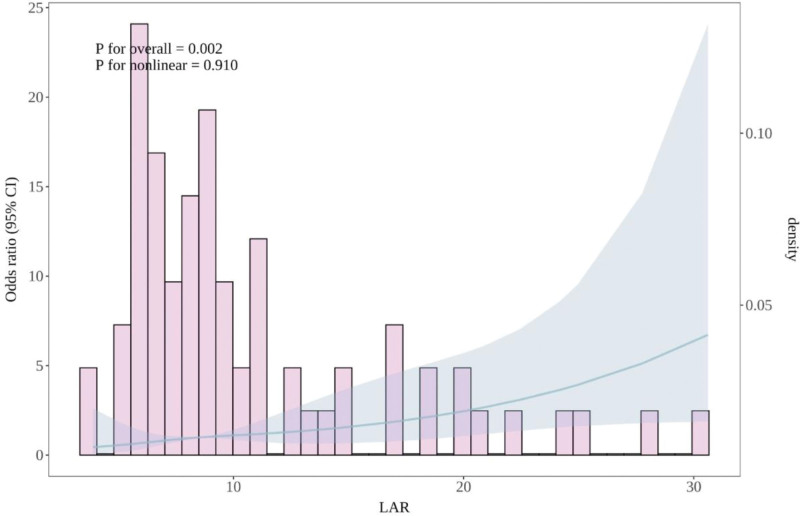
Relationships between the LAR and the risk of 28-d all-cause mortality in patients with septic shock. CI = confidence interval, LAR = lactate dehydrogenase-to-albumin ratio.

**Figure 3.**
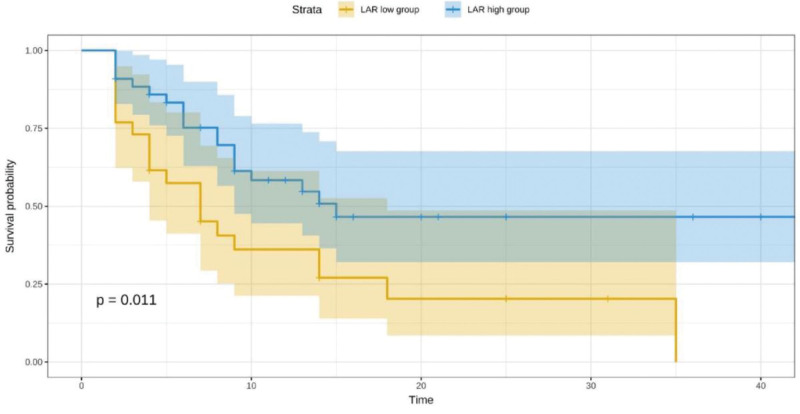
Kaplan–Meier survival curve of the 28-d cumulative survival rate of patients with septic shock.

Based on the RCS-defined cutoff, patients were stratified into a high-LAR group (≥10.1) and a low-LAR group (<10.1). Kaplan–Meier survival analysis showed that the high-LAR group had significantly lower 28-day cumulative survival compared to the low-LAR group (*P* = .011; Figs. 2 and 3).

## 4. Discussion

In this retrospective study of 93 patients with septic shock, we found that the lactate dehydrogenase-to-albumin ratio (LAR) and WBC count were independent predictors of 28-day mortality. Patients with a high LAR (≥10.1) had significantly lower survival compared to those with a low LAR. RCS analysis further demonstrated a nonlinear association between LAR and mortality risk. These findings suggest that LAR, a simple biomarker derived from routine laboratory tests, can provide useful prognostic information for critically ill patients with septic shock.

Our findings are consistent with previous studies using the MIMIC-IV database, which also demonstrated that LAR is an independent predictor of mortality in septic patients.^[2,8]^ Sun et al confirmed its prognostic value in septic shock,^[2]^ while Guan et al reported a nonlinear association between LAR and all-cause mortality in ICU patients with sepsis.^[9]^ By validating these observations in a Chinese cohort, our study adds to the evidence that LAR may be a broadly applicable marker across different populations.

Building on these comparative findings, it is important to consider the biological basis of the LAR. Albumin (ALB), the most abundant plasma protein, is synthesized in the liver and constitutes approximately 50% to 60% of total plasma proteins in healthy adults.^[7,10]^ It has a half-life of about 21 days^[7,8]^ and is negatively charged at physiological pH. Beyond its role in maintaining colloidal osmotic pressure, albumin also possesses antioxidant, anti-inflammatory, transport, endothelial-stabilizing, and immunomodulatory properties.^[11]^ Albumin synthesis depends on nutritional status and is influenced by proinflammatory cytokines such as tumor necrosis factor alpha and interleukin-6, which can suppress protein synthesis.^[12]^ This explains the frequent occurrence of hypoalbuminemia in patients with septic shock. Albumin stability and recycling are mediated by the neonatal Fc receptor, which is expressed by hepatocytes and endothelial cells.^[13]^ In the later stages of septic shock, widespread microcirculatory dysfunction and capillary endothelial damage reduce neonatal Fc receptor expression, further compromising albumin homeostasis.

LDH is a ubiquitous intracellular enzyme involved in glycolysis and is present in a wide range of organisms, including bacteria, plants, and animals.^[14]^ It is a known marker of tissue injury and has been widely studied in various diseases, including cancer.^[15,16]^ In our study, LDH levels were elevated in both the survival and mortality groups, but the difference between the 2 groups was not statistically significant. This suggests that LDH alone may not be a reliable predictor of mortality in patients with septic shock.

The lactate dehydrogenase to albumin ratio (LAR) has been shown to correlate with outcomes in malignancies^[17–19]^ and has recently gained attention in sepsis research. However, relevant studies remain limited. A recent study^[9]^ based on the MIMIC IV database reported a nonlinear association between LAR and all cause mortality in ICU patients with sepsis, suggesting its potential utility as a prognostic biomarker, although it did not specifically analyze septic shock. Sun et al^[2]^ further explored this correlation and confirmed the prognostic value of LAR in patients with septic shock using the same database. In our study, several variables were significantly higher in the mortality group, including age, APACHE II score, SOFA score, LAR, lactic acid, ICU length of stay, WBC count, and BUN. Moreover, the use of mechanical ventilation and norepinephrine was more common in the mortality group, reflecting more severe illness and complex clinical management. These findings are consistent with clinical observations. Notably, norepinephrine use was recorded as a binary variable rather than by dose. Previous studies have suggested that a supratherapeutic dose of norepinephrine may be harmful, but the optimal threshold remains controversial.^[20]^ Timing of administration is also critical, with studies showing that a 1 hour delay in norepinephrine initiation increases mortality by 5.3%.^[21]^ Future research should include the dose and timing of norepinephrine use to provide a more comprehensive analysis.

Multivariate analysis identified LAR and WBC count as independent predictors of mortality. The optimal LAR cutoff of 10.1 was associated with significantly increased risk, offering clinicians a simple indicator for prognosis. Early identification of patients with elevated LAR could support timely intervention and tailored management. Norepinephrine findings should be interpreted cautiously. We recorded norepinephrine use only as a binary variable, without dosing or timing. Previous studies have shown that supratherapeutic dosing may worsen outcomes^[20]^ and that each 1-hour delay in initiation increases mortality by 5.3%.^[21]^ Future studies should capture dose and timing to clarify this relationship.

This study has several limitations. First, it was a retrospective analysis conducted in a single center with a relatively small cohort, which may limit the generalizability of the findings. Second, norepinephrine use was recorded only as a binary variable, without information on dose or timing, which restricted our ability to fully evaluate its prognostic significance. Third, we were unable to perform receiver operating characteristic curve analyses to quantify the discriminative performance of LAR and WBC due to dataset restrictions. These limitations should be considered when interpreting our results, and larger multicenter prospective studies are warranted to validate and expand on these findings.

## 5. Conclusion

This study confirms that the lactate dehydrogenase-to-albumin ratio (LAR) is an independent predictor of 28-day mortality in patients with septic shock. A LAR ≥ 10.1 was associated with significantly lower survival, highlighting its value as a simple and accessible biomarker for early risk stratification. However, the study has important limitations, including its retrospective single-center design and relatively small cohort, which may restrict the generalizability of the findings. Larger prospective, multicenter studies are needed to validate these results and determine whether incorporating LAR into risk assessment can improve patient outcomes.

## Author contributions

**Conceptualization:** Yin-Yin Wu, Li Yao.

**Data curation:** Yin-Yin Wu.

**Formal analysis:** Yin-Yin Wu.

**Investigation:** Yin-Yin Wu.

**Methodology:** Yin-Yin Wu.

**Supervision:** Li Yao.

**Writing** – **original draft:** Yin-Yin Wu, Li Yao.

**Writing** – **review & editing:** Li Yao.
